# Rapid visualization of grain boundaries in monolayer MoS_2_ by multiphoton microscopy

**DOI:** 10.1038/ncomms15714

**Published:** 2017-06-05

**Authors:** Lasse Karvonen, Antti Säynätjoki, Mikko J. Huttunen, Anton Autere, Babak Amirsolaimani, Shisheng Li, Robert A. Norwood, Nasser Peyghambarian, Harri Lipsanen, Goki Eda, Khanh Kieu, Zhipei Sun

**Affiliations:** 1 Department of Electronics and Nanoengineering, Aalto University, Tietotie 3, Espoo FI-02150, Finland; 2 Institute of Photonics, University of Eastern Finland, P.O. Box 111, Joensuu FI-80101, Finland; 3 Department of Physics, University of Ottawa, 25 Templeton Street, Ottawa, Ontario, Canada K1N 6N5; 4 College of Optical Sciences, University of Arizona, 1630 East University Boulevard, Tucson, Arizona 85721, USA; 5 Department of Physics, National University of Singapore, 2 Science Drive 3, Singapore 117551, Singapore

## Abstract

Grain boundaries have a major effect on the physical properties of two-dimensional layered materials. Therefore, it is important to develop simple, fast and sensitive characterization methods to visualize grain boundaries. Conventional Raman and photoluminescence methods have been used for detecting grain boundaries; however, these techniques are better suited for detection of grain boundaries with a large crystal axis rotation between neighbouring grains. Here we show rapid visualization of grain boundaries in chemical vapour deposited monolayer MoS_2_ samples with multiphoton microscopy. In contrast to Raman and photoluminescence imaging, third-harmonic generation microscopy provides excellent sensitivity and high speed for grain boundary visualization regardless of the degree of crystal axis rotation. We find that the contrast associated with grain boundaries in the third-harmonic imaging is considerably enhanced by the solvents commonly used in the transfer process of two-dimensional materials. Our results demonstrate that multiphoton imaging can be used for fast and sensitive characterization of two-dimensional materials.

## Abstract (web summary)

Atomically thin transition metal dichalcogenides can be grown on large scale using chemical vapour deposition which, however, determines presence of grain boundaries. Here, the authors report that third-harmonic generation imaging provides excellent sensitivity and fast speed for grain boundary visualization in MoS_2_.

Since the discovery of graphene, two-dimensional (2D) layered materials, such as molybdenum disulphide (MoS_2_), have been studied extensively for various applications^1^,^2^, ranging from transistors^3^, light emitters^4^,^5^,^6^ and photodetectors^7^ to modulators^8^. A key requirement for these 2D material applications is the development of industrial-scale, reliable, inexpensive production processes, while providing a balance between ease of fabrication and final material quality. Chemical vapour deposition (CVD) is one of the promising pathways to fabricate high-quality and relatively large-area 2D materials^2^,^9^,^10^. Even though the quality of mechanically exfoliated samples is usually better than that of CVD grown ones, it is evident that compared with the exfoliation method, CVD has superior potential as a large-scale fabrication technique for future industrial applications of 2D materials. Thus, development of tools and methods to examine the properties and quality of CVD-grown MoS_2_ and other 2D materials is extremely important. Typically, as-fabricated CVD MoS_2_ samples consist of irregularly shaped individual grains forming a discontinuous monolayer. Therefore, methods that can discriminate crystal orientation as well as identify grain boundaries (GBs) are particularly valuable. In particular, fast methods to characterize GBs and fabricated samples have become important for inspecting quality of large-scale samples^11^,^12^,^13^,^14^,^15^, which are needed for various future industrial applications.

Typically, the GBs in graphene and MoS_2_ have been comprehensively studied using transmission electron microscopy (TEM)^16^,^17^,^18^, which gives detailed information about the crystal structure at the GBs. However, TEM is time-consuming, expensive and requires the use of a special substrate. In addition, the size of the characterization area is limited to the nanometre scale in the TEM method. Photoluminescence (PL) imaging has been proposed to visualize the GBs in MoS_2_ (refs 19, 20). However, our results show that the PL imaging is not very sensitive to the GBs when there is a small crystal axis rotation between the neighbouring grains. Raman microscopy has been typically used for characterizing the quality of the MoS_2_, as well as the number of layers. The Raman mapping has also been reported to visualize GBs but showing only small signal shift (∼one wavenumber) on both Raman modes (e.g. 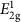 and *A*_1g_) on the GBs^19^. It is noteworthy that Raman and PL microscopies are typically very slow measurement techniques due to the need to collect a wide spectrum within a specific wavelength range (depending on the band gap of the materials, Raman excitation wavelength and the locations of the Raman modes) at each measurement point, which reduces their applicability for characterizing large area samples.

During the last few years, the nonlinear optical properties of transition metal dichalcogenides have gained a lot of interest^13^,^14^,^15^,^21^,^22^. In contrast to even numbers of MoS_2_ layers, odd numbers of layers have been shown to generate second-harmonic signals due to the broken inversion symmetry in the crystal structure^13^,^14^,^15^. Therefore, second-harmonic generation (SHG) has been proposed to resolve the crystal orientations and GBs in MoS_2_ crystals^11^,^14^,^21^. However, this is not an effective method for probing the GBs when the crystal axis rotation between the neighbouring grains is small. Furthermore, SHG can only be used for determining the crystal orientation of flakes with an odd number of layers, as MoS_2_ flakes with an even number of (A-B stacked) layers do not produce SHG due to their centrosymmetric crystal structure. Recently, thin (∼5 nm^22^) and monolayer^23^,^24^ MoS_2_ flakes have been reported to enable third-harmonic generation (THG) with infrared excitation, with relatively high conversion efficiency. In addition, even high harmonic generation has been recently reported from a monolayer MoS_2_ (ref. 25). Further, there have been several recent reports about the effects of chemical doping on the optical and electronic properties of MoS_2_ (refs 26, 27, 28, 29). These studies show that both chemical doping by various solvents and laser treatment can significantly modify the PL of monolayer MoS_2_. However, the effect of chemical doping on the nonlinear optical properties of 2D materials is still completely unexplored.

In this work, the nonlinear optical properties of CVD-grown MoS_2_ are characterized by multiphoton (SHG and THG) microscopy using excitation wavelength of 1,560 nm. In contrast to Raman-, PL- and SHG-based characterization methods, we demonstrate that THG can be effectively used to study the GBs in CVD MoS_2_. To compare the existing and the proposed linear and nonlinear optical techniques with each other, we characterize the same flake with Raman, PL and multiphoton microscopies. Measurement setups and conditions are described in detail in Methods. Compared with the polarization sensitive SHG technique, our proposed THG method is based on a simpler experimental setup and provides excellent sensitivity in observing GBs. Our multiphoton characterization method is also approximately four orders of magnitude faster compared with the Raman and PL imaging where the spectrum of generated optical signal needs to be collected over a wide wavelength range at every measurement point. In addition to demonstrating that THG microscopy can be employed for visualizing the GBs, we also show that the combination of solvents and laser scanning dramatically improves the contrast of the detected THG signal between the GBs and the grains. We have measured effective third order susceptibilities,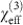, for as-grown monolayer CVD MoS_2_ (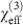
=1.2 × 10^−19^ m^2^ V^−2^) and after the chemical treatment (∼ 3.0 × 10^−19^ and 3.7 × 10^−19^ m^2^ V^−2^ on grains and on GBs, respectively).

## Results

### Multiphoton characterization

**Figure 1:**
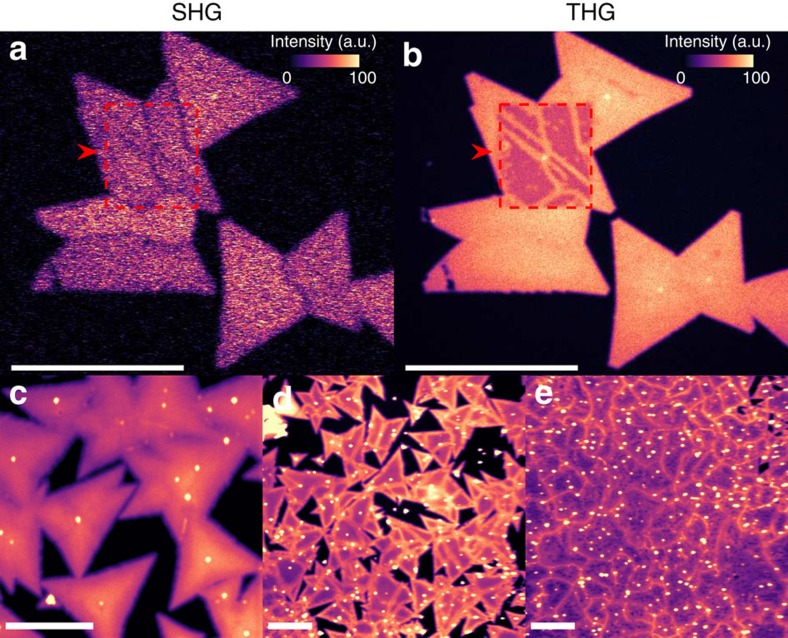
Multiphoton imaging of CVD-grown large-area MoS_2_ flakes. (**a**) SHG and (**b**) THG images of Sample 1. An area of ∼25 × 30 μm^2^ is first exposed (marked by a red dashed border) by scanning over it with the same laser used for the multiphoton laser-scanning microscopy. The laser beam had a fluence of 21 mJ cm^−2^ and scanning speed was 20 μs per pixel. After exposure, the SHG and THG images have been captured using a laser fluence of 11 mJ cm^−2^. In **a**, the GBs with larger crystal orientation mismatch are visible in SHG images, which show no visible difference between the exposed and unexposed areas. In **b**, all the GBs are clearly visible in THG images on the exposed area. Similar THG images of (**c**) as-grown MoS_2_ sample without any post treatments and (**d**,**e**) as-grown MoS_2_ samples after ACE+IPA treatment. The GBs appear bright after ACE+IPA treatment. The laser fluence in **c**–**e** was ∼177 mJ cm^−2^. Scale bars, 25 μm (**a**,**b**) and 50 μm (**c**–**e**).

We carried out studies on different MoS_2_ flakes and observed that the THG signal changes after chemical treatment and laser illumination, depending on the fluence of the incident laser. All of the SHG and THG images shown here are created by scanning a laser beam (spot size ∼1.85 μm) over the sample with two galvo mirrors and recording the counts from two photomultiplier tubes at each position of the laser beam. See Methods section for details. Figure 1a,b presents SHG and THG images of Sample 1. The square with a red dashed border shows an area that has been first irradiated by our multiphoton microscope using a 1,560 nm excitation laser with a fluence of 21 mJ cm^−2^. After that, the whole area (Fig. 1a,b) was measured with the same excitation laser but with a lower fluence of 11 mJ cm^−2^ using the same microscope. In the THG image (Fig. 1b), a clear difference between areas that were and were not exposed to higher fluence laser can be observed. The GBs are not visible in the area that was irradiated only with lower fluence (see the area outside of the red dashed box in Fig. 1b). We see that the THG signal has decreased on the grains inside the area that has received a pre-exposure to higher fluence excitation laser before imaging. However, there is no THG signal decrease on the GBs and therefore the GBs can be seen as bright lines inside the illuminated area (the red dashed box in Fig. 1b). In the SHG image (Fig. 1a), the exposed area is not observable, indicating that the SHG signal is not affected by the high fluence laser pre-exposure. The origin of the SHG signal is in the asymmetric crystal structure of MoS_2_ and therefore the unchanged SHG signal suggests that the actual MoS_2_ layer is not affected by the laser exposure. Therefore, we believe that the THG signal on the GBs and the grains that were irradiated during the image scanning only with lower fluence, is enhanced by some adsorbed molecules on the surface. These molecules may be removed from the grains but not from the GBs by laser irradiation, which results in the observed decrease in the THG signal on grains but not on GBs. This is reasonable, because the GBs contain more dislocations and defects that can act as strong adsorption sites for molecules^18^. These adsorbed molecules are present during a typical transfer process where the polymethyl methacrylate (PMMA) is removed using acetone (ACE) + isopropanol (IPA) treatment (see Discussion section for more details).

To further study the effects of the chemical treatment (ACE+IPA) and the laser exposure, we performed similar measurements on as-grown and chemically treated flakes with even larger fluence. Figure 1c shows a THG image of an as-grown MoS_2_ sample, which is not transferred or chemically treated. THG images in Fig. 1d,e were measured from an as-grown MoS_2_ sample that was first treated 30 min in ACE and then followed by a 5 min IPA treatment. After that, the sample was blown dry using compressed air and imaged with our multiphoton microscopy with a fluence of ∼177 mJ cm^−2^. The GBs are not visible in Fig. 1c, showing that the THG signal on the GBs is identical to that on grains without chemical treatment. However, after ACE+IPA treatment, the GBs become clearly visible in the THG images (Fig. 1d,e, fluence of ∼177 mJ cm^−2^). Therefore, we further conclude that increased THG signal on GBs is due to ACE+IPA treatment and laser irradiation. The image sizes are 300 μm × 300 μm in Fig. 1d,e. We see that multiphoton microscopy is capable of large area sample characterization.

**Figure 2:**
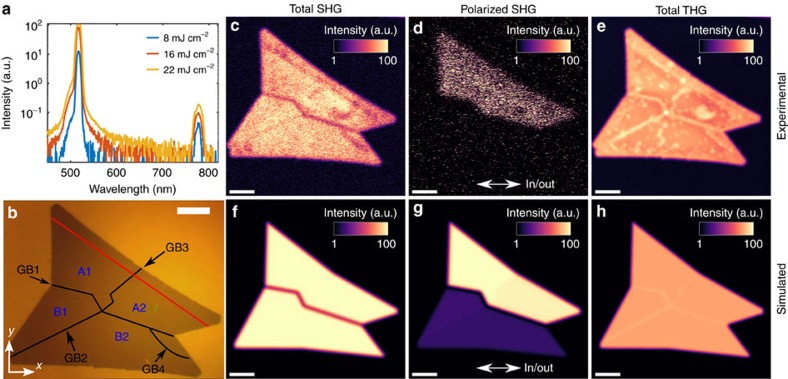
Multiphoton characterization results. (**a**) Generated multiphoton spectra with different input fluences, (**b**) optical image with marked grains (A1, A2, B1 and B2) and grain boundaries (GB1, GB2, GB3 and GB4), (**c**) experimental SHG image without analyser, (**d**) experimental SHG image with parallel polarized excitation and detection (as indicated by a double-headed arrow), (**e**) experimental THG image without analyser, (**f**) simulated SHG image without analyser, (**g**) simulated SHG image with parallel polarized excitation and detection (as indicated by a double-headed arrow) and (**h**) simulated THG image without analyser. Scale bars, 10 μm. Colour scale bars are the counts from photomultiplier tubes.

The output spectra obtained with the multiphoton microscopy from CVD grown MoS_2_ (ACE+IPA treated) for various fluences of the excitation laser are plotted in Fig. 2a. The graph shows two peaks corresponding to second- (780 nm) and third-harmonic (520 nm) generated signals using the excitation wavelength of 1,560 nm. Interestingly, the THG signal is approximately two orders of magnitude stronger than the SHG signal at the excitation wavelength of 1,560 nm. Part of this can be explained by resonance enhancement, as the THG is on-resonance and SHG is off-resonance. The effect of resonance enhancement can be approximated with the classical anharmonic oscillator model^30^, as the nonlinear susceptibilities are proportional to the linear susceptibilities both at the fundamental and harmonic frequencies. The absolute value of the permittivity of MoS_2_ at 520 nm is ∼1.8 times larger than that at 780 nm^31^,^32^. Therefore, using the anharmonic oscillator model one indeed expects the near-resonance THG susceptibility at 520 nm to be ∼1.8-fold enhanced compared with off-resonant THG susceptibility at 780 nm. This argumentation also agrees with the experimental finding discussed in ref. 14 that the effect of the resonance enhancement on SHG signal efficiency in MoS_2_ is approximately one order of magnitude. When the resonance enhancement and the incident light intensity are fully considered, and using previously measured nonlinear material parameters^30^,^31^,^32^, we estimate that the experimental THG/SHG intensity ratio in our experiments with an incident beam power of 10 mW (fluence of around 8 mJ cm^−2^) at 1,560 nm should be on the order of 2 × 10^−3^. This intensity ratio is proportional to the incident beam power, but even for a considerably higher incident beam power of 100 mW (fluence of 80 mJ cm^−2^) the calculated ratio is only around 2 × 10^−2^. If the effect of resonance enhancement is neglected, the estimated THG/SHG ratio would be on the order of 7 × 10^−4^. We note that the calculated THG/SHG intensity ratio, after taking resonant enhancement into account, is still small because in general THG is a much weaker process compared with SHG. Thus, even when the resonance enhancement of the third-order susceptibility is taken into account, THG should still be considerably smaller than SHG. We measure a THG/SHG intensity ratio of ∼270, which is over 4 orders of magnitude larger than what would be expected by the classical anharmonic oscillator model. Therefore, we conclude that the observed THG/SHG ratio cannot be explained solely through the effect of resonant enhancement using existing material parameters and the classical anharmonic oscillator model. Our recent study suggests that the high THG/SHG ratio can be explained by the low-energy band structure of MoS_2_, when trigonal warping of the energy bands is taken into account^23^. At low photon energies (here ∼0.8 eV) the SHG only probes the low-energy band structure, which is nearly rotationally invariant with only trigonal warping that reduces the full rotational symmetry to a three-fold rotational symmetry^23^. In another recent study high harmonic generation in monolayer MoS_2_ was studied with infrared excitation (0.3 eV)^25^. The authors report similarly higher intensity for odd harmonics compared with neighbouring even harmonics for higher order harmonics from 6th to 13th harmonic.

However, we must emphasize that the main point is not the high THG but rather the high contrast ratio, induced by the combination of ACE+IPA treatment and laser exposure, between the THG from the grains and GBs.

We have quantified the effective bulk-like third order susceptibility 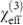 –values of pristine MoS_2_ and after ACE+IPA treatment. We employed a method similar to that in ref. 23 using exfoliated monolayer graphene on a similar substrate as a reference sample. The 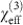 –values were obtained from measurements shown in Fig. 1c,d using the following equation


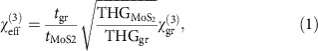


where *t*_gr_=0.33 nm is the thickness of graphene and 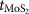
=0.65 nm is the thickness of monolayer MoS_2_. THG_gr_ and 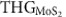 are measured THG signals from graphene and MoS_2_, respectively. We used the reference value of 3.25 × 10^−19^ m^2^ V^−2^ for the effective third-order susceptibility of graphene (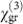)^33^ and obtained 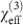
=1.2 × 10^−19^ m^2^ V^−2^ for as-grown CVD MoS_2_. After chemical treatment, 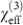 increases to ∼ 3.0 × 10^−19^ m^2^ V^−2^ on grains and 3.7 × 10^−19^ m^2^ V^−2^ on GBs. Using these values we can now understand the results shown in Fig. 1b. Before laser exposure, the chemically treated flakes contain a uniform layer of adsorbed molecules, which increases the overall THG response, compared with as-grown flakes. After laser exposure, the adsorbed molecules are removed from grain areas, reducing the measured THG signal. However, the molecules remain adsorbed near GBs, giving rise to the contrast to visualize the GBs. Our experimental values of 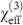 are in agreement with previously reported values. In a recent study, effective third-order susceptibility of 2.4 × 10^−19^ m^2^ V^−2^ was reported for CVD-grown monolayer MoS_2_ (ref. 24), which agrees well with our values. In ref. 22, the authors report a value on the order of ∼10^−19^ m^2^ V^−2^ for seven-layer-thick mechanically exfoliated samples, which is close to the values measured here, even though our samples were CVD-grown monolayers. Furthermore, we have also measured the effective bulk-like second order nonlinear susceptibility by using similar method as in ref. 23. It is worth noting that our measured effective second-order susceptibility |*χ*^(2)^| of monolayer MoS_2_ is ∼ 2 pm V^−1^, which is comparable to the previously reported values at 1,560 nm excitation^34^,^35^.

An optical wide-field image of a sample is shown in Fig. 2b highlighting the observed grains and GBs as discussed later. SHG and THG images obtained without an analyser in front of the detector are shown in Fig. 2c,e, respectively. Both of the images are acquired simultaneously without using an analyser and therefore indicate the total generated harmonic signals. The horizontal dark line in the middle of the flake is clearly observable in the SHG image and is attributed to GB1 (cf. Fig. 2b); however, GB2 and GB3 cannot be observed in the SHG image. The SHG signal was reported earlier to decrease on the GB due to the destructive interference of the SHG fields from the neighbouring grains, explaining why GB1 appears dark in the image^21^.

In contrast to the SHG results, the GBs appear bright in the THG image and all the GBs 1–4 can be clearly observed in the THG image. For example, GB2 from the left bottom corner of the flake is seen as brightly as GB1 in the middle of the flake (see Fig. 2e), whereas the GB2 is barely visible in the corresponding SHG image (see Fig. 2c). Another bright line (GB3) in the THG image is also observed to go from the middle of the top edge to the nucleation point in the middle of the flake where all the bright lines combine (bright dot in the THG image, Fig. 2e). The THG signal on the GBs is ∼15% stronger than that on the grains (see Supplementary Note 3 for details). A line scan over the GB is presented in Supplementary Fig. 3. The THG signal is also slightly increased near the edges of the flake.

Figure 2d presents results for SHG imaging where a polarizer has been inserted right before the focusing objective making the polarizations of the input and output beams parallel. Using this parallel-polarized excitation and detection, one can resolve the MoS_2_ crystal orientations (details are presented in Supplementary Note 1, Supplementary Movies 1 and 2 and Supplementary Figs 1 and 2)^13^,^14^,^15^. Only the area above GB1 is visible in Fig. 2d, suggesting a large difference in the crystal orientations between the upper (A1 and A2) and lower (B1 and B2) parts. It also shows that the crystal axis rotation between the grains A1 and A2, as well as between the grains B1 and B2, may be too small to be observable with the parallel-polarized SHG measurement.

In SHG microcopy, the contrast difference between neighbouring grains is due to the anisotropic polarization pattern of SHG^13^,^14^,^15^. The GBs can also be seen in SHG images as dark lines due to destructive interference of SHG from neighbouring grains^11^,^21^. The occurrence of destructive interference depends on whether the edges of the grains are Mo- or S-terminated. Destructive interference occurs when the edges of the neighbouring grains are of the same type and the incident light polarization is perpendicular to the bisecting direction of the grain edges^11^. In general, all GBs cannot be directly visualized in a single SHG image. Instead, several images with different angles between the polarization of the incident light and the crystal axis need to be taken to visualize the GBs. In contrast, with THG microscopy, after chemical treatment, all GBs can be directly seen from one image without the need of any further data analysis, which makes THG imaging a faster and more convenient method for imaging GBs. Moreover, SHG can only be utilized to study flakes with an odd number of layers since MoS_2_ crystals with an even number of layers do not emit SHG. THG does not depend on the parity of the layer number, thus it can be used for characterization of flakes with either an even or odd number of layers.

### Simulations of SHG and THG in MoS_2_ flakes

The SHG and THG effects in monolayer MoS_2_ flakes are simulated using an approach based on free-space Green's functions and the Rayleigh–Gans approximation described in detail in Methods section. The image of the simulated total SHG is shown in Fig. 2f. GB1 is clearly visible in the simulated image and corresponds very well with the experimental results (see Fig. 2c), as other GBs are not observed. The decreased SHG on GB1 is verified to be due to the destructive interference of the SHG fields from neighbouring grains. In the case of GB2 and GB3, the crystal axis rotation between the neighbouring grains is small and therefore the GBs behave as if there was a single uniform grain.

The simulated SHG image for parallel-polarized excitation and detection is presented in Fig. 2g. This also shows the strongest SHG from grains A1 and A2 as observed in the experimental image (see Fig. 2d). Therefore, theory and experiment are in a good agreement in the case of SHG. Our physical understanding of the situation suggests that the third-order nonlinearity of the material should be higher on the GBs than on the grains due to the adsorbed molecules from ACE+IPA treatment. When including a slightly higher (10% higher) third-order nonlinearity coefficient on the GBs, the simulated image matches well with the experimental image. The THG signal image shown in Fig. 2h is calculated without using an analyser. In addition, we performed simulations for cases where the analyser is placed before the objective lens and the incident polarization is rotated. As expected based on symmetry arguments, the THG signals were found to be independent of the polarization angle as was also observed in the experiments. This suggests that the THG method is simpler than the SHG approach.

### Raman characterization

**Figure 3:**
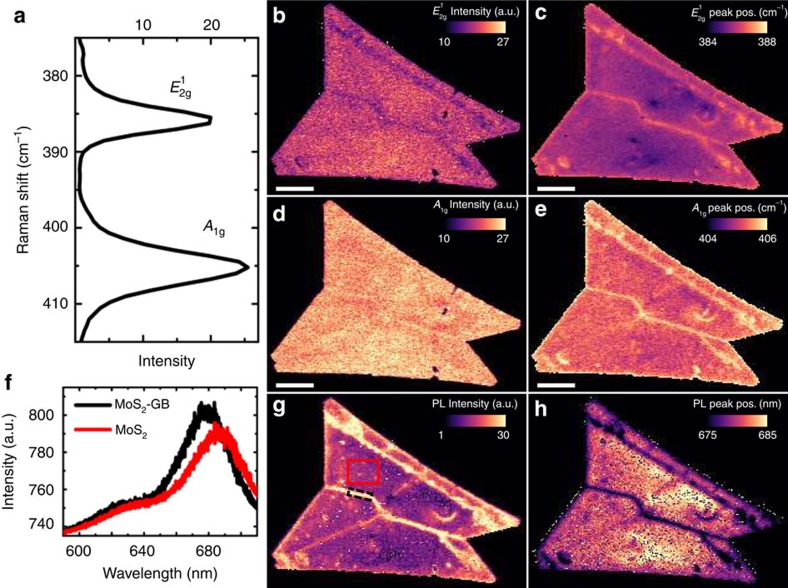
Raman and photoluminescence characterization. (**a**) Raman spectrum, (**b**) intensity of 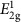 peak, (**c**) centre position of 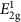 peak, (**d**) intensity of *A*_1g_ peak and (**e**) centre position of *A*_1g_ peak, (**f**) PL spectra from the middle of the grain A1 (red curve, taken from the area marked with white line in **g** and from GB1 in the middle of the flake (black curve, taken from the area marked with a black dashed line in **g**. (**g**) PL intensity image of the 680 nm peak and (**h**) centre position of the 680 nm peak. Intensities and peak positions are acquired by Gaussian fitting. Scale bars, 10 μm. Colour scale bars are in CCD counts (Raman and PL intensities), in cm^−1^ (Raman peak positions) and in nm (PL peak position).

The Raman spectrum of the CVD-grown MoS_2_ is plotted in Fig. 3a. Peaks are observed at Raman shifts of ∼386 and ∼405 cm^−1^, corresponding to the in-plane 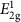 and the out of plane 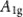 modes, respectively^36^,^37^. The Raman intensity map of the 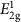 peak (the sample used in Fig. 2) is shown in Fig. 3b and the peak position map is shown in Fig. 3c. GB1 in the middle of the flake appears bright in the Raman peak position (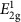) image. The 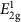 mode stiffens (∼1 wavenumber shift in the Raman peak from 385.5 to 386.5 cm^−1^) on this GB. In the Raman intensity image, the same line appears slightly darker than the other areas. The Raman intensity image of the *A*_1g_ peak is shown in Fig. 3d (the centre position image in Fig. 3e). Furthermore, the *A*_1g_ mode noticeably stiffens (∼1 wavenumber shift, from 405.2 to 406.1 cm^−1^) on GB1 (bright horizontal line in Fig. 3e). The slight increase in the *A*_1g_ peak intensity can be observed on the GBs. The 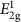 mode softens (from 305.5 to 304.8 cm^−1^) and the *A*_1g_ mode stiffens (from 405.2 to 408.5 cm^−1^) at the nucleation point.

### Photoluminescence characterization

Photoluminescence characterization is carried out with an excitation wavelength of 532 nm and with an average power of ∼100 μW. The PL characterization results for the sample (the flake used in Fig. 2) are also shown in Fig. 3. The PL spectra of CVD-grown MoS_2_ measured from the middle of the grain (B2) and from GB1 are plotted in Fig. 3f. Two PL peaks are observed around 680 and 630 nm, corresponding to the A and B direct excitonic transitions in MoS_2_, respectively^3^. The PL intensity and center position images of the 680 nm peak are shown in Fig. 3g,h, respectively. The PL intensity and centre positions are acquired by fitting a Gaussian lineshape to the peak at 680 nm. The PL intensity is enhanced at the GBs (∼3 times stronger on GB1 compared with the signal on grain B2). We also observed that the PL peak blue-shifts on the GBs (from 685 nm (1.81 eV) to 674 nm (1.837 eV) corresponding to an energy shift of 27 meV). The intensity increase and peak shift are clear on GB1. However, intensity is only slightly increased on GB2 and GB3. Neither is the peak shift clearly observed on these two GBs.

Earlier, the increased PL intensity and the blue-shifted peak have been reported on oxygen defect engineered and chemically *p*-doped MoS_2_ (refs 38, 39). Our studies suggest that the ACE+IPA treatment can be used to chemical doping of the GBs. We also studied the PL properties of other pristine MoS_2_ flakes not exposed to the multiphoton laser beam and observed that the PL intensity is always enhanced and blue-shifted on GBs with large crystal axis rotation. This verifies that the PL characteristics of the sample are not due to laser irradiation during the multiphoton characterization. Typical PL results for these other areas are detailed in Supplementary Note 4 and shown in Supplementary Fig. 4.

## Discussion

Different grains and GBs of the sample are schematized in the optical microscope image in Fig. 2b. GB1 is clearly observable in the Raman shift of the 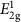 and *A*_1g_ peaks, PL intensity, SHG and THG images. The same GB appears dark and bright in the non-polarized SHG (Fig. 2c) and THG images (Fig. 2e), respectively. The polarized SHG (Fig. 2d) shows that the upper and lower parts have a large relative rotation of their crystal axes. However, we cannot observe any difference in the SHG signal between grains A1 and A2, nor between B1 and B2. This suggests the same or almost same crystal orientation exists in these areas. The GBs between A1 and A2 as well as between B1 and B2 can only be observed with good confidence level with PL and THG, with the latter providing better contrast.

We suggest that the enhancement of the PL and THG signals is due to the doping caused by the ACE+IPA treatment. The results show that crystal axis rotation mismatch at the GBs has a clear impact on the PL shift, as well as on the strength of the PL. The PL peak is only slightly shifted at GB2 and GB3 (small, <1 degree, crystal axis rotation between neighbouring grains, see Supplementary Fig. 2) in contrast to GB1 (large, ∼28° crystal axis rotation, see Supplementary Fig. 2). Similar PL intensity increase and blue-shift due to the oxygen defects and chemical doping have been reported earlier^38^,^39^ and our findings on the GBs are in a good agreement with these earlier results. Furthermore, our THG results also imply that with high intensity laser beam exposure we remove some molecules from the grains but not from the GBs. This suggests that these molecules are more strongly bound at the dislocations on the GBs. The remaining adsorbed molecules can then affect the overall THG efficiency by two possible mechanisms. First, the molecules will have a non-negligible third-order response and will therefore themselves also emit some THG. Second, adsorbed molecules will also change the electronic properties of MoS_2_ layer itself, increasing the THG efficiency of the layer. Therefore, we strongly believe that the increased and blue-shifted PL on the GBs is indeed due to chemisorbed molecules (for example, leftovers from ACE+IPA treatment), which also play a key role in the stronger THG signal on the GBs.

We conclude that the GBs with a large relative crystal axis rotation between the neighbouring crystal structures are clearly observable in the Raman shifts of the 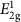 and *A*_1g_ peaks, and in the PL intensity and peak position images, as well as in the SHG and THG images. All GBs are clearly visible in the THG image of the flake (see Fig. 2e). However, our theoretical model does not predict any enhancement of the THG signal on the GBs due to the crystal orientation mismatch (as is the case with reduced SHG on GB1). Therefore, we conclude that the *χ*^(3)^ of the overall material must be somehow modified at the GBs. Based on the other experimental results (Fig. 1b), we strongly believe this modification to be due to the adsorbed molecules from the ACE+IPA treatment. This hypothesis also agrees reasonably well with the very recent finding that electrons can transfer from solvents to MoS_2_ layers, causing significant changes in their physical properties^26^. However, surprisingly, the effect caused by the laser exposure seems to give rise to negligible changes in SHG efficiency (see Fig. 1a). It must be noted here that we do not have sufficient measurement results to reliably explain the origin of the more intense THG on GBs, which require further study. However, we do believe that this important observation opens up a whole new area of research concerning the effect of chemical doping and laser exposure on the nonlinear optical properties of MoS_2_ and other 2D materials.

Finally, we discuss the advantages of using THG microscopy for visualizing GBs. The nonlinear optical analysis of the grain structures, conducted via measuring SHG and THG signals, is approximately four orders of magnitude faster compared with the Raman and PL techniques: the former requires few seconds, whereas the latter two techniques easily require hours of data acquisition. For example, full Raman mapping (WITec Alpha300 R) of the sample consists of 210 × 180 pixels (70 × 60 μm^2^) (Fig. 3b–e). The Raman spectrum was recorded in each of the pixels and the measurement time of a single Raman spectrum per pixel was 0.2 s. Thus, the total time to measure the Raman map is 210 × 180 × 0.2 s=7,560 s, which equals 2 h and 6 min. For comparison, the THG and SHG images here consist of 1,000 × 1,000 pixels (260 × 260 μm^2^) and the acquisition rate was 20 μs per pixel, yielding a total measurement time of 20 s. Based on these values, the image acquisition time for THG and SHG images is around four orders of magnitude smaller than that for Raman mapping. Further, it is possible to use THG microscopy to visualize GBs between grains, which have only a small crystal axis rotation between the grains. This is not always possible with Raman measurements. For example, with THG microscopy we can detect GBs (GB2 in Fig. 2b) between grains that have only ∼0.4° difference between the crystal axes, which cannot be seen from the Raman images. Worth noting, our nonlinear characterization result also shows that the THG signal in MoS_2_ is about two orders of magnitude stronger than SHG at the excitation wavelength of 1,560 nm and shows the highest contrast for GBs, indicating that THG can be a very sensitive method for characterizing 2D layered materials.

In conclusion, we used simultaneous SHG and THG imaging methods to study the crystal orientations and the GBs of CVD-grown MoS_2_ flakes. We demonstrated that THG is sensitive to solvents commonly used in the transfer process of CVD-grown MoS_2_ samples. We measured the effective bulk-like third order susceptibility of as-grown CVD MoS_2_ to be 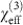
=1.2 × 10^−19^ m^2^ V^−2^ and found that after the chemical treatment the 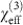 increases to ∼3.0 × 10^−19^ m^2^ V^−2^ on grains and 3.7 × 10^−19^ m^2^ V^−2^ on GBs. This results in a change of contrast between the THG emitted from grains and GBs, which can be utilized for visualization of GBs. We have shown that GBs with large relative crystal axis rotation between neighbouring crystal structures are clearly observable with Raman, PL, SHG and THG imaging. However, we found that THG imaging is a superior method for distinguishing GBs with little relative rotation of the crystal axes, much more sensitive than PL and SHG approaches. Further, our multiphoton characterization results demonstrate that THG imaging is approximately four orders of magnitude faster than Raman and PL mapping, and therefore more suitable for high-volume and large-size sample characterization.

## Methods

### CVD growth of MoS_2_

Monolayer MoS_2_ crystals were grown by an atmospheric pressure thermal CVD method. A horizontal tube furnace with 1 inch-diameter quartz tube was used. MoO_3_ powder (30 mg) was placed in a ceramic boat and the SiO_2_/Si substrate was faced down and mounted on top of the boat. A separate ceramic boat with sulfur powder (50 mg) was placed next to the MoO_3_ powder in an upstream position. During the synthesis of MoS_2_ crystals, the reaction chamber was heated to 700 °C in 50 s.c.c.m. Ar and kept there for 5 min for MoS_2_ crystal growth. The fabricated flakes are then transferred on another SiO_2_/Si substrate using PMMA. The PMMA is then removed by a 30 min ACE treatment followed by a 5 min IPA treatment (ACE+IPA treatment).

### Multiphoton microscopy

The nonlinear optical properties of the CVD-grown MoS_2_ samples were investigated using a multiphoton microscope, described in more detail in the Supplementary Note 5 and shown schematically in Supplementary Fig. 5. The design of the microscope and the experimental procedure were previously reported^40^,^41^,^42^. The laser beam is scanned with a 2D galvo mirror system and focused on the sample using a × 20 microscope objective with numerical aperture of 0.5. The focal spot size, measured by nonlinear razor blade method, is ∼1.8 μm.

Two different mode-locked erbium-doped fiber lasers operating at a central wavelength of 1,560 nm can be used as a light source in the system. The lasers have different repetition rates and thus produce different peak powers. For the lower peak power laser, the maximum average power on the sample is 30 mW with a repetition rate of ∼50 MHz and ∼150 fs pulse duration at the sample surface, yielding an estimated pulse peak power of ∼4 kW, pulse energy of 0.6 nJ and fluence of 22 mJ cm^−2^. The lower peak power laser was used for Figs 1a,b and 2a–c,e. The parameters of the higher peak power laser are: 38 mW, 8 MHz, 100 fs, 47 kW, 4.8 nJ and 177 mJ cm^−2^. The higher peak power laser was used for Fig. 1c,d,e.

The backscattered SHG and THG signals generated from each point on the sample are guided to the detection arm by an 870 nm dichroic mirror and split into two paths using a long-pass dichroic mirror (cutoff at 562 nm) and finally detected using photomultiplier tubes. Narrow band-pass filters are used to select SHG and THG signals at central wavelengths of 780 and 520 nm, respectively. The acquisition of the two channels is simultaneous, making the measurement conditions for both channels exactly identical regardless of any perturbations (external vibrations or fluctuations in laser power). For resolving the light spectrum, the generated light is guided to a spectrometer (OceanOptics QE PRO-FL) by rotating the 870 nm dichroic mirror. A polarizer can be added to the setup before the microscope objective yielding parallel-polarized excitation and generated light.

### SHG and THG microscopy simulations

Numerical simulations of the multiphoton microscopy were performed using an approach based on free-space Green's functions and Rayleigh–Gans approximation^43^. The symmetry of the MoS_2_ flakes on glass substrate was assumed to belong to *D*_3h_ point group as previously reported^14^. Therefore, for the THG process the non-zero and independent susceptibility components reduce to *zzzz*, *xxyy*, *xxzz* and *zzxx*, where *x* and *y* point along the flake surface (as shown in Fig. 2b) and *z* is perpendicular to the surface^30^. For SHG process, there is only one independent component *yyy*^14^,^30^. The model MoS_2_ flake was constructed to consist of four grains with varying crystal orientations similar to the flake shown in Fig. 2b. Using polarized SHG optical images (see details in the Supplementary Note 2 and in Supplementary Fig. 2), the crystal orientations were estimated to be 38.8°, 38°, 6.7° and 7.1° for the grains B2, B1, A2 and A1, respectively, where a 0 (90)° would point along the *x* direction (*y* direction). An example numerical SHG microscopy image is shown in Fig. 2f where the total collected SHG image clearly shows only the GB1 where the crystal orientation difference is largest. When an analyser is parallel to the linear polarization of the input beam, clear dependence of the SHG signal on the grains crystal orientations is found (see Fig. 2g). An example numerical THG image without using any analyser is shown in Fig. 1h.

To account for the effect of having adsorbed molecules on the GBs for THG, the third-order nonlinearity coefficient was increased by 10% on the GBs. This order-of-magnitude increase was estimated by using known literature values for the macroscopic third-order susceptibility χ^(3)^∼10^−19^ m^2^ V^−2^ (ref. 22) and estimating the atomic number density of the MoS_2_ monolayer to be ∼3 × 10^30^ m^−3^ (ref. 44). The second-order hyperpolarizability was then calculated to be ∼3.33 × 10^−49^ m^5^ V^−2^. For simplicity, we next assumed that the adsorbed molecules were ACE molecules, but similar order-of-magnitude estimates could similarly be reached for other molecules. The second-order hyperpolarizability values for ACE and for other small molecules can be reasonably well estimated using the bond-charge model^30^. When the effects of bond orientations on the resulting hyperpolarizability are neglected, we calculate a hyperpolarizability value of ∼3 × 10^−50^ m^5^ V^−2^ for ACE. Therefore, the 10 % increase of the effective macroscopic third-order susceptibility of the MoS_2_ monolayer due to adsorbed ACE molecules per MoS_2_ unit cell is a reasonable order-of-magnitude estimation. This increase also agrees with our experimental finding.

### Raman and PL characterization

Raman and PL spectra were acquired by a Witec micro-Raman spectrometer equipped with 1,800 and 600 lines per mm gratings, respectively, and excited with a Nd:YAG laser emitting at 532 nm. The power on the sample was approximately 0.1 mW for PL and Raman measurements. Scattered light was collected through a × 100 objective. The Raman and PL intensity and peak positions were acquired by Gaussian fitting.

### Data availability

The data that support the findings of this study are available from the corresponding author upon reasonable request.

## Additional information

**How to cite this article:** Karvonen, L. *et al*. Rapid visualization of grain boundaries in monolayer MoS_2_ by multiphoton microscopy. *Nat. Commun.* **8,** 15714 doi: 10.1038/ncomms15714 (2017).

**Publisher's note:** Springer Nature remains neutral with regard to jurisdictional claims in published maps and institutional affiliations.

## Supplementary Material

Supplementary Information Supplementary Figures, Supplementary Notes and Supplementary References

Supplementary Movie 1 Changes in the intensity of SHG with alternating polarization direction. The excitation is at 1040 nm and the excitation and detection are parallel-polarized. The crystal orientations shown in Fig. 2b are obtained from this data. The SHG signal intensities with altering polarization from different grains are also shown in the Supplementary Fig. 2b.

Supplementary Movie 2 A movie created from a series of SHG images with parallel polarized excitation-detection. Each frame corresponds to different polarization of the excitation beam. The intensity differences between grains with differing crystal orientations can be clearly seen. Snapshots from this movie can be seen in Supplementary Fig. 1.
