# Supplementary material for: Rapid visualization of grain boundaries in monolayer MoS2 by multiphoton microscopy
Source: Nat Commun. 2017 Jun 5;8:15714. doi: 10.1038/ncomms15714 (PMC5465365; doi:10.1038/ncomms15714)
Supplement: Supplementary Information — Supplementary Figures, Supplementary Notes and Supplementary References [file ncomms15714-s1.pdf]

Supplementary Note 1. Resolving crystal orientations

The second-harmonic generation (SHG) images of CVD grown molybdenum disulphide (MoS_2) flakes measured with different polarization directions are shown in Supplementary Figure 1. The excitation and detection beams are parallel-polarized. The SHG intensity difference between different grains is due to the crystal orientations[1]. The polarization angle is rotated 10 degree between adjacent images. The images are similar after 60 degree polarization rotation (e.g. see images in the top left corner and the second rightmost on the bottom row) as expected from the symmetry[1]. This shows that polarized SHG imaging is a powerful tool for resolving the crystal orientations in MoS_2.

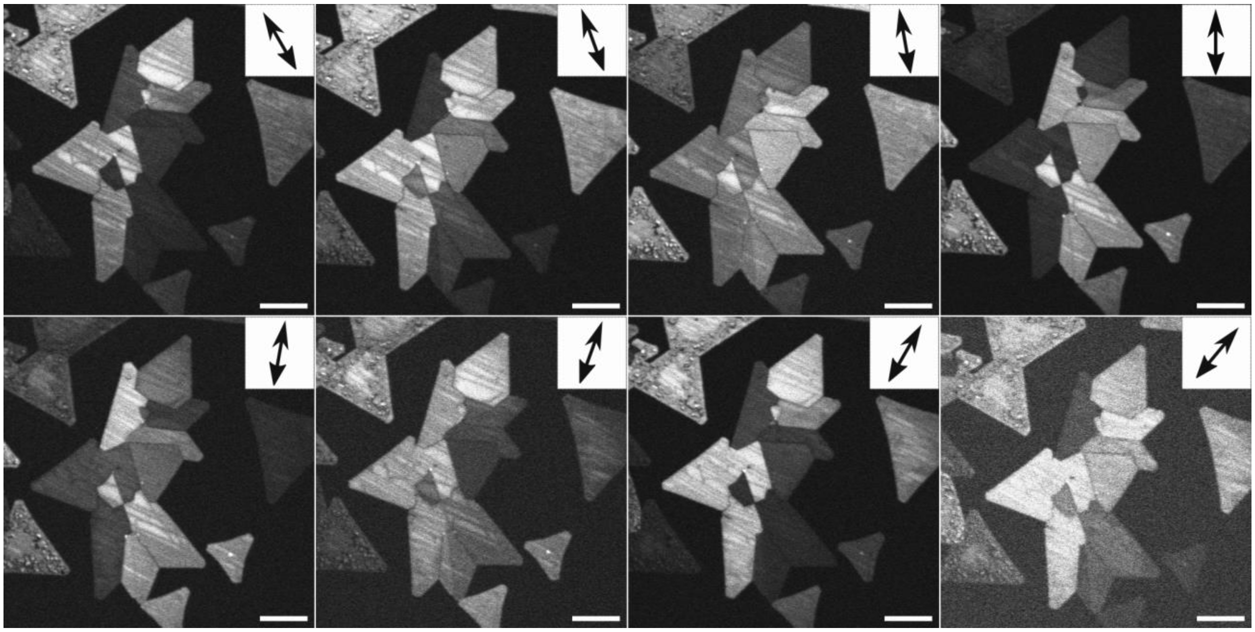

Supplementary Figure 1 SHG images of the MoS_2 flake measured with different polarization angles. The measurement is done with 1040 nm excitation laser (fluence ~177 mJ cm^{-2}). The excitation and detection are parallel-polarized. Black arrows in inset figures show the polarization direction. The polarization rotation is 10 degree between the images. The images look similar after every 60 degree polarization rotation as expected from the theory[1]. Scale bars are 20 μm.

Supplementary Note 2. Crystal orientations in Sample 2

Supplementary Figure 2 represents the results of the crystal orientation characterization of Sample 2 performed similarly as in Figure 1. Grains are marked with different colors and names into the optical wide-field image of Sample 2 in Supplementary Figure 2a. Dark lines are schematizing the GBs as characterized in the main article. Fig. S2b shows the polarization dependency of the SHG signal from different grains (A1, A2, B1 and B2). The polarization angles (parallel-polarized excitation and detection) correspond to the coordinate system shown in Supplementary Figure 2a. Solid lines are sine fits to the measurement points. Sine fits also resolves the crystal orientation differences between the grains giving the values of 38.8, 38, 6.7 and 7.1 degrees for A1, A2, B1 and B2, respectively. If we mark B1 to zero, then the crystal orientations of the grains A1, A2 and B2 compared to B1 are 27.9, 28.7 and 0.4 degrees, respectively.

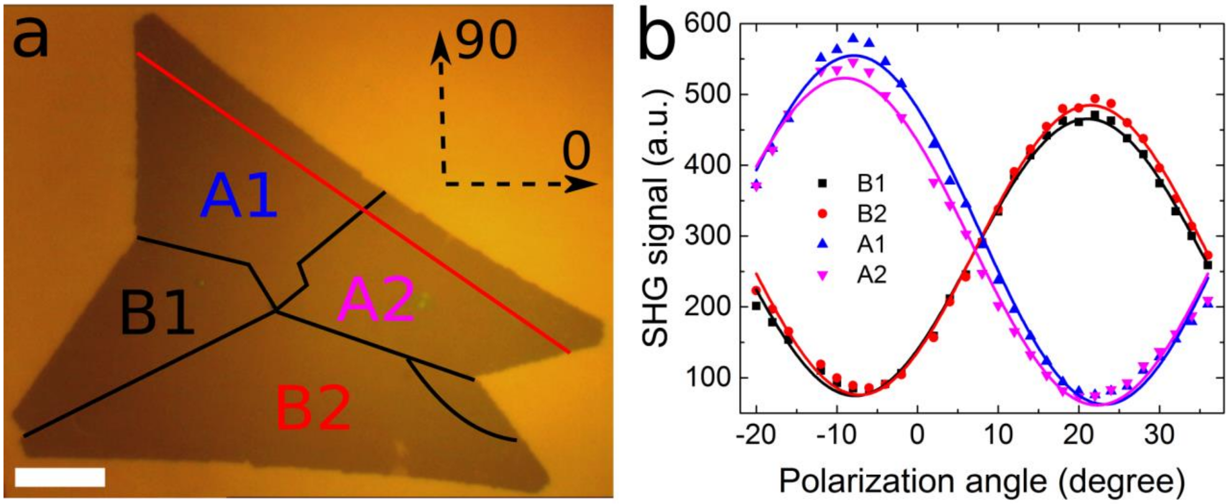

Supplementary Figure 2 Resolving the crystal structure of Sample 2. a) Grains marked (A1, A2, B1 and B2) to optical wide-field image of Sample 2. b) SHG signal from different grains as a function of polarization angle (parallel-polarized excitation and detection). Solid lines are sine fits to the measured SHG signals. Polarization angle refers to the coordinates shown in a). 0 angle means x-axis in the SHG

and optical images. SHG signal is measured with 1040 nm excitation laser (fluence ~177 mJ cm^{-2}). Scale bar is 10 μm.

Supplementary Note 3. Second- and third-harmonic generated signal differences on GBs

The Supplementary Figure 3a presents the RGB composite image from MoS_2 flake studied in the article. The SHG signal appears red and the third-harmonic generated (THG) signal as green in the image. The SHG and THG cross-sections are plotted in Supplementary Figure S3b taken from the black dashed line in Supplementary Figure 3a. The cross-sections show about 15% increase of THG signal and about 50% decrease of SHG signal on the GB.

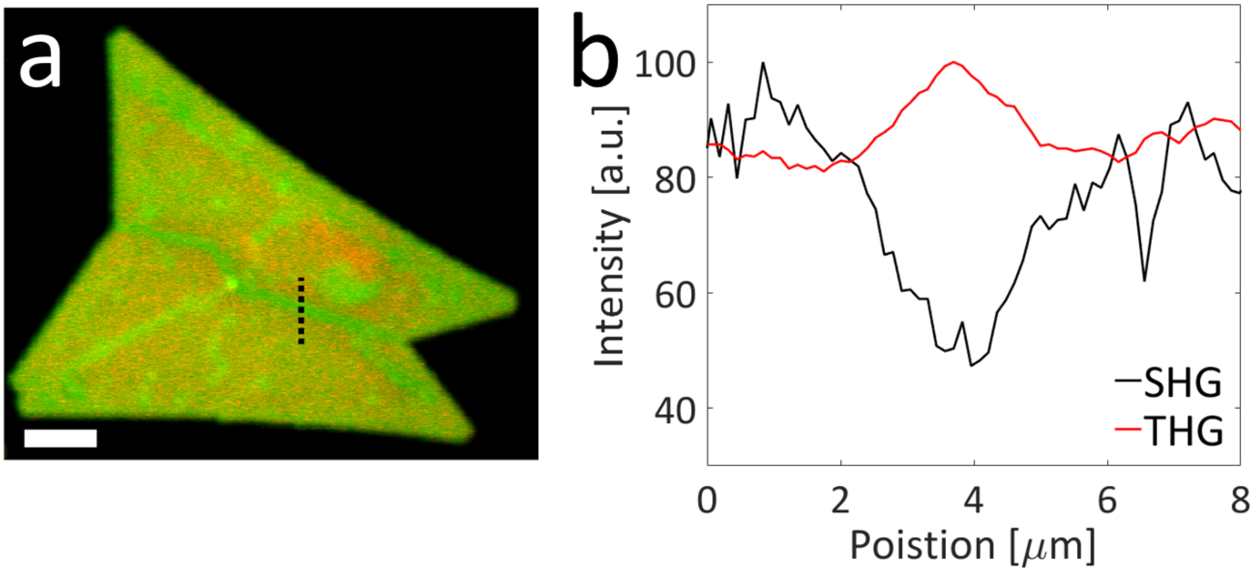

Supplementary Figure 3 Composite SHG and THG image. a) The RGB composite image constructed from SHG and THG images. SHG appears red and THG as green in the image. b) Cross-sectional plot of SHG (black curve) and THG (red curve) signals taken from the black dashed line in (a). Both SHG and THG signals have been normalized separately so that maximum value is 100. Scale bar is 10 μm. A 1560 nm laser with fluence of 21 mJ cm^{-2} was used for recording the image in Supplementary Figure 3a. The THG and SHG curves in b) have been normalized for comparison of the signal strengths over the grain boundary.

Supplementary Note 4. Photoluminescence characterization

Photoluminescence (PL) intensity and peak position images of three different MoS_2 flakes are shown in Supplementary Figure 4. The flake in Supplementary Figures 4a and 4d was not irradiated by an intense fs-laser beam (used for the multiphoton characterization) whereas the flake in Supplementary Figures 4b and 4e was irradiated prior to the PL characterization. Both flakes give similar PL signals, verifying that the PL peak position or intensity differences between grains and grain boundaries are not due to the laser irradiation during multiphoton characterization.

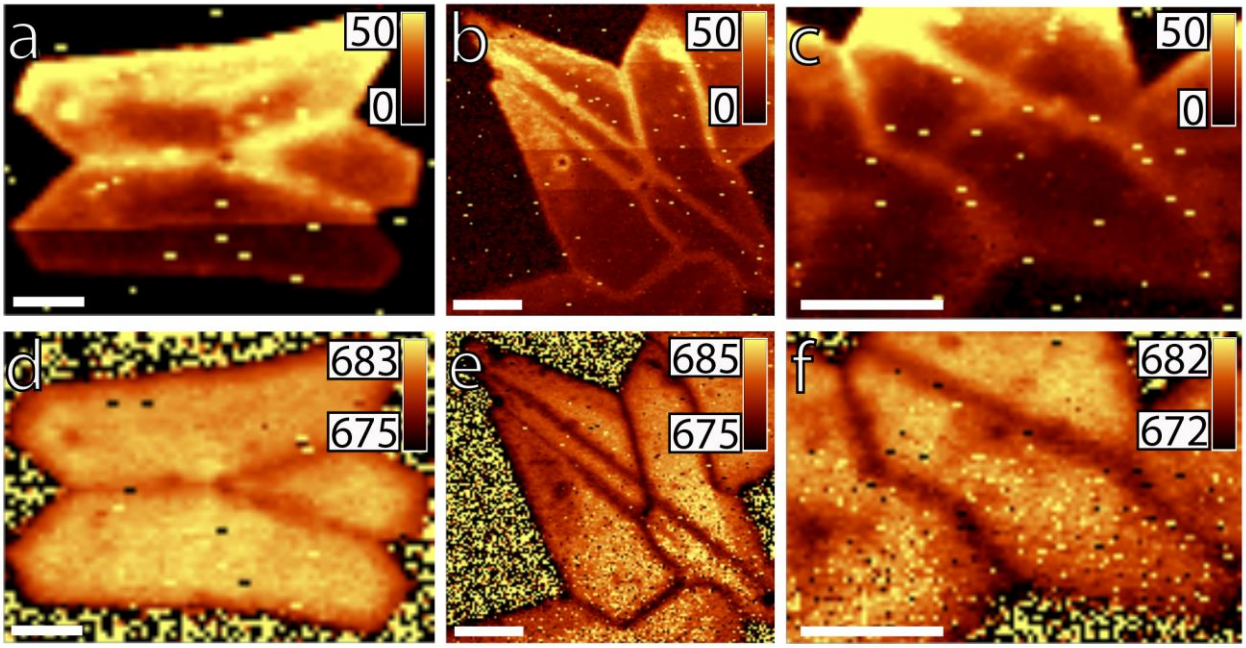

Supplementary Figure 4 Photoluminescence and Raman characterization. Photoluminescence images a)-c), PL intensity images and d)-f), PL (peak position) of different MoS_2 flakes showing enhanced and blue-shifted PL on the GBs. The flake in a) and d) was not irradiated by an intense fs-laser beam, whereas the flake (the illuminated area in Figure 1) in b) and e) was irradiated during multiphoton characterization prior to the PL characterization. The flake (the pristine area in the right bottom corner in Figure 1) in c) and f) is only moderately illuminated during multiphoton microscopy. Scale bars in the images are 10 μm.

Supplementary Note 5. Multiphoton microscope

Schematic of the multiphoton microscope is shown in Supplementary Figure 6. MLL refers to the mode-locked fiber laser. The fiber lasers with the wavelengths of 1560 nm and 1040 nm are used in the results shown in Supplementary Information. At center wavelength of 1560 nm, two lasers with different repetition rates can be used, the higher repetition rate is ~50 MHz and lower is ~8 MHz. The average output power of 1560 nm laser with a repetition rate of ~50 MHz (R) is 56 mW leading to the maximum average power of 30 mW (P_{avg}) and ~150 fs (τ) pulse duration at the sample surface, yielding an estimated pulse peak power of ~4 kW (P_p), pulse energy of 0.6 nJ (E_p) and fluence of 22 mJ cm^{-2} (f). With 1560 nm and 8 MHz laser, the parameters are: P_{avg} = 38 mW, τ = 100 fs, E_p = 4.8 nJ, P_p = 47 kW and f = 177 mJ cm^{-2}.

The average output power of the 1040 nm excitation laser (only used for Supplementary Figures 1 and 2) is 75 mW leading to the average power of 38 mW on the sample. With 1040 nm laser the parameters are: R = 8 MHz, P_{avg} = 38 mW, τ = 150 fs, E_p = 4.8 nJ, P_p = 32 kW, f = 177 mJ cm^{-2}.

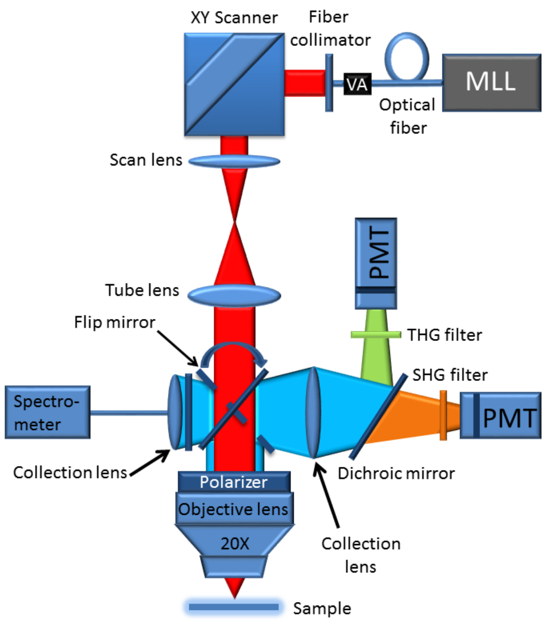

Supplementary Figure 5 Schematic diagram of the multiphoton microscope. MLL refers to mode-locked fiber laser, VA variable attenuator and PMT photomultiplier tube.

Supplementary References

1. Li, Y. *et al.*, Probing symmetry properties of few-layer MoS_2 and h-BN by optical second-harmonic generation. *Nano Lett.* **13**, 3329-3333 (2013).
